# Xylanase Supplementation Modulates the Microbiota of the Large Intestine of Pigs Fed Corn-Based Fiber by Means of a Stimbiotic Mechanism of Action

**DOI:** 10.3389/fmicb.2021.619970

**Published:** 2021-03-24

**Authors:** Amy L. Petry, John F. Patience, Nichole F. Huntley, Lucas R. Koester, Michael R. Bedford, Stephan Schmitz-Esser

**Affiliations:** ^1^Department of Animal Science, Iowa State University, Ames, IA, United States; ^2^Iowa Pork Industry Center, Iowa State University, Ames, IA, United States; ^3^Department of Veterinary Microbiology and Preventive Medicine, Iowa State University, Ames, IA, United States; ^4^AB Vista Feed Ingredients, Marlborough, United Kingdom

**Keywords:** gut microbiota, insoluble fiber, stimbiotic, swine, xylanase, mucosa, colon, cecum

## Abstract

This research tested the hypothesis that xylanase modulates microbial communities within the large intestine of growing pigs fed corn-based fiber through a stimbiotic mechanism(s) of action (MOA). Sixty gilts were blocked by initial body weight, individually housed, and randomly assigned to one of four dietary treatments (*n* = 15): a low-fiber (LF) control, a high-fiber (HF) control containing 30% corn bran, HF+100 mg/kg xylanase (HF+XY), and HF+50 mg/kg arabinoxylan-oligosaccharide (HF+AX). Pigs were fed dietary treatments for 46 days. On day 46, pigs were euthanized, and mucosa and lumen contents were collected from the cecum and the colon. The V4 region of 16S rRNA genes was sequenced and clustered into 5,889, 4,657, 2,822, and 4,516 operational taxonomic units (OTUs), in the cecal contents and mucosa and colonic contents and mucosa, respectively. In cecal contents, HF+XY increased measures of *α*-diversity compared to LF (*p* < 0.001). Relative to LF, HF increased the prevalence of 44, 36, 26, and 8, and decreased 19, 9, 21, and 10, of the 200 most abundant OTUs from the cecal contents and mucosa and colonic contents and mucosa, respectively (*Q* < 0.05). Compared to LF, HF increased the abundance of OTUs from the *Treponema_2*, *Ruminococcus_1* genera, from the *Lachnospiraceae*, *Ruminococcaceae*, and *Prevotellaceae* families. In contrast, relative to LF, HF decreased *Turicibacter* and *Lactobacillus* in the cecal contents, and *Megasphaera* and *Streptococcus* in the mucosa. Relative to HF, HF+XY increased 32, 16, 29, and 19 and decreased 27, 11, 15, and 10 of the 200 most abundant OTUs from the cecal contents and mucosa and colonic contents and mucosa, respectively (*Q* < 0.05). The addition of xylanase to HF further increased the abundance of OTUs from the *Lachnospiraceae* and *Ruminococcaceae* families across the large intestine. Compared to HF, HF+XY increased the abundance of *Lactobacillus*, *Bifidobacterium*, and *Faecalibacterium* among all locations (*Q* < 0.05). However, HF+AX did not increase the prevalence of these genera in the large intestine. Supplementing xylanase to HF increased hidden-state predictions of microbial enzymes associated with arabinoxylan degradation, xylose metabolism, and short-chain fatty acid production. These data suggest xylanase elicits a stimbiotic MOA in the large intestine of pigs fed corn-based fiber.

## Introduction

It is well established that pigs do not synthesize the endogenous enzymes required to digest dietary fiber (DF). Thus, pigs must rely on their gastrointestinal microbiota to ferment DF into metabolizable substrates (Varel and Yen, 1997). Modern swine diets are trending to contain increased concentrations of DF. This is often a consequence of mitigating rising feed costs by utilizing economically-priced industrial co-products. Many of these co-products fed to pigs are corn-based, and often shift the carbohydrate composition of the diet to favor DF, rather than starch (Acosta et al., 2020). It is well known that increasing DF can reduce nutrient and energy digestibility, impair hindgut fermentation, and decrease pig performance (Gutierrez et al., 2013; Weber et al., 2015). The DF innate to corn, and its co-products, is almost entirely insoluble and largely composed of arabinoxylans (Jaworski et al., 2015). Corn-based arabinoxylans are poorly fermented by resident microbiota due to their structural complexity and poor solubility (Bach Knudsen, 2014). One potential strategy to improve the fermentability of DF, and possibly mitigate its negative effects, is to include exogenous carbohydrases into the diet.

Xylanase is a logical carbohydrase to include in corn-based diets as it hydrolyzes the β-(1-4) glycosidic bonds of arabinoxylans (Dodd and Cann, 2009). Furthermore, it may improve DF fermentation by depolymerizing arabinoxylans into lower molecular weight fragments that are more soluble and fermentable (Pedersen et al., 2015). Indeed, xylanase often improves the nutrient and fiber digestibility of corn-based diets in the large intestine of the pig (Petry et al., 2019). Microbial fermentation largely occurs in the cecum and colon due to the dense microbial populations in these gastrointestinal regions (Jensen and Jørgensen, 1994). Additionally, there are increasing reports that supplementation of xylanase to swine benefits markers of improved gastrointestinal health, and commonly reduces finishing pig mortality in commercial production (Petry and Patience, 2020). These improvements in health are often postulated to result from modulation of microbial populations in the gut. However, there is a paucity of research investigating the composition of hindgut microbiota in pigs fed corn-based fiber supplemented with xylanase with a long adaptation period. Moreover, the *in vivo* mechanism(s) of action (MOA) of xylanase in the presence of corn-based DF has yet to be fully elucidated.

The *in-situ* release products of xylanase are likely arabinoxylan oligosaccharides (AXOS), and high molecular weight arabinoxylan polysaccharides. It has been reported that these AXOS can be rapidly fermented by the resident microbiota and modulate the gastrointestinal microbiota in the large intestine of poultry *via* a stimbiotic MOA (Bautil et al., 2020; González-Ortiz et al., 2020). However, there is a dearth of research in swine (Petry and Patience, 2020). A stimbiotic was defined by González-Ortiz et al. (2019), as “*an additive that stimulates a fiber-degrading microbiome resulting in an increase in fiber fermentability even though the additive itself contributes little to short chain fatty acid production*.” The proposed mechanism of a stimbiotic is that it accelerates the capacity of the large intestine to more efficiently digest fiber by modulating microbial taxa that produce their own carbohydrases (Bautil et al., 2020; González-Ortiz et al., 2020). Therefore, the experimental objective was to characterize the modulation of hindgut microbiota due to increased corn-based fiber, xylanase, and directly-supplemented AXOS in pigs fed corn-based diets for 46 days. We hypothesized that the addition of xylanase or AXOS would modulate intestinal microbial communities to more effectively support fiber degradation through a stimbiotic MOA.

## Materials and Methods

### Animals, Diets, and Experimental Design

The investigation into the gastrointestinal microbiota reported herein is in continuation of related research reported by Petry et al. (2020a,b). Readers are referred to Petry et al. (2020a) for greater detail on animal methods and diet compositions. Methods reported herein are adapted from Petry et al. (2020a), and are provided to briefly orient readers to the study design. All analytical methods exclusive to these data reported herein are provided.

Sixty growing gilts with an initial body weight of 25.4 ± 0.9 kg were used in three replicates (20 gilts per replicate) of a 46-day trial. Gilts were blocked by initial body weight and randomly assigned within a block to one of four dietary treatments: a low-fiber (LF) control with 7.5% neutral detergent fiber (NDF), a high-fiber (HF) control with 30% corn bran without solubles (NDF = 21.9%), HF+100 mg xylanase/kg (HF+XY; Econase XT 25P; AB Vista, Marlborough, United Kingdom) providing 16,000 birch xylan units per kg, and HF+50 mg arabinoxylan-oligosaccharide/kg (HF+AX; 3–7 degrees of polymerization). Pigs were individually housed and fed *ad libitum* for 36 days to provide adequate time for the pig and their microbiota, to adapt to dietary treatments. On day 36, pigs were moved to metabolism crates for a 10-day metabolism study reported by Petry et al. (2020a). During the metabolism study, pigs were limit fed 80% of the average daily feed intake determined based on the intake of the first replicate averaged across all treatments. The daily feed allotment was split into two feedings. Pigs were given *ad libitum* access to water throughout the duration of the total study.

### Sample Collection

On day 46, pigs were fed half of their total daily feed allotment, and after consumption, were euthanized by captive bolt stunning and exsanguination. Pigs were necropsied for collection of lumen contents and mucosa from the cecum and colon. Approximately, 30 ml of cecal contents was collected, subsampled, snap-frozen in liquid nitrogen, and stored at −80°C pending DNA extraction. The apex of the cecum was isolated and rinsed with sterile phosphate buffered solution to remove remnants, and mucosal scrapings were carefully collected from the tissue, snap-frozen in liquid nitrogen, and stored at −80°C pending DNA extraction. Similarly, contents from the ascending portion of the spiral colon were collected, subsampled, and snap-frozen in liquid nitrogen, and stored at −80°C. An approximately 4-cm long section proximal to the ascending spiral colon was isolated and flushed with sterile phosphate buffered solution. The colonic tissue was scraped for mucosa, and the scrapings were snap-frozen in liquid nitrogen and stored at −80°C pending DNA extraction.

### DNA Extraction and 16S rRNA Gene Illumina MiSeq Sequencing

Total genomic DNA was extracted from cecal and colonic mucosa and contents using a DNeasy PowerLyzer PowerSoil Kit (Qiagen, Germantown, MD) according to the manufacturer’s instructions. Extracted genomic DNA concentration and purity were evaluated using a ND-1000 spectrophotometer (NanoDrop Technologies, Rockland, DE), and subsequently stored at −80°C for later sequencing. All samples had 260:280 nm ratios above 1.84. Extracted DNA was adjusted to a total well concentration of 125 ng of DNA, and sequencing was conducted by the DNA facility at Iowa State University (Ames, IA).

PCR-amplified 16S rRNA gene sequencing was conducted using a previously established protocol designed to amplify bacteria and Arcaea (Caporaso et al., 2018). Concisely, one replicate per sample of extracted genomic DNA was amplified using Platinum™ Taq DNA Polymerase (Thermo Fisher Scientific, Waltham, MA). Mutual 16S rRNA bacterial primers 515F (5'-GTGYCAGCMGCCGCGGTAA-3'; 21) and 806R (5'-GGACTACNVGGGTWTCTAAT-3'; 22) for the variable region V4 were utilized as previously described (Kozich et al., 2013). All samples underwent PCR with an initial denaturation step at 94°C for 3 min, followed by 35 PCR cycles (45 s of denaturing at 94°C, 20 s of annealing at 50°C, and 90 s of extension at 72°C), and concluded with a 10 min extension at 72°C. The subsequent PCR products were purified with a QIA quick 96 PCR Purification Kit (Qiagen Sciences Inc., Germantown, MD) according to the manufacturer’s directions. The bar-coded amplicons were included at equivalent molar ratios and used for Illumina MiSeq paired-end sequencing with 250 bp read length and cluster generation with 10% PhiX control DNA on an Illumina MiSeq platform (Illumina Inc., San Diego, CA).

### Sequence Analyses and Prediction of Functional Potential

Once sequencing was complete, corresponding overlapping paired-end reads was stitched to obtain an ultimate amplicon size of 255 bp. The sequencing data for each sample were screened for quality, and paired-end reads were combined using mothur (v.1.40.4). Sequences that contained ambiguous bases, were shorter than 250 bp, longer than 255 bp, contained homopolymers >8 bases in size, and potential chimeric sequences were removed. Remaining sequences were then clustered into operational taxonomic units (OTUs) with a 99% sequence similarity based on a distance matrix generated in mothur. Consensus taxonomy for OTUs was assigned using version 132 of the SILVA SSU database (Pruesse et al., 2007). Shannon, Simpson, and Chao1 *α*-diversity indices were calculated as previously described (Kim et al., 2017).

The hidden-state predictions of gene families and their abundance within a given location were constructed using the 2.3.0b version of Phylogenetic Investigation of Communities by Reconstruction of Unobserved States 2 (PICRUSt2), according to default parameters (Douglas et al., 2020). Briefly, representative 16S rRNA gene amplicon sequences for each OTU were aligned and placed into a phylogenetic tree with relation to reference genomes from the Integrated Microbial Genomes database using the provisions of HMMER, EPA-ng, and GAPPA (Barbera et al., 2019; Bengtsson-Palme et al., 2020; Czech et al., 2020).

Hidden-state gene predictions were determined by castor references based on nearest-sequenced taxon index, with a threshold of 2 (Louca and Doebeli, 2018). The hidden-state predictions of 2,381 enzymes were characterized from the Enzyme Commission (EC) database (Caspi et al., 2018). A total of 25 enzymes associated with arabinoxylan degradation, pentose metabolism, or short chain fatty acid (SCFA) production were selected (Table 1), normalized by predicted 16S rRNA gene copy number per OTU, and subsequentially analyzed using preplanned contrasts.

**Table 1:** Enzymes associated with arabinoxylan degradation, pentose metabolism, or short chain fatty acid (SCFA) production that were selected for gene prediction-based analysis using Phylogenetic Investigation of Communities by Reconstruction of Unobserved States 2 (PICRUSt2).

Enzyme Commission (EC) number	Accepted name
1.1.1.9	D-xylulose reductase
1.1.1.27	L-lactate dehydrogenase
1.1.1.28	D-lactate dehydrogenase
2.3.1.8	Phosphate acetyltransferase
2.7.2.1	Acetate kinase
2.7.2.7	Butyrate kinase
2.7.2.15	Propionate kinase
2.7.1.17	Xylulose kinase
2.8.3.1	Propionate CoA-transferase
2.8.3.8	Acetate CoA-transferase
2.8.3.9	Butyrate-acetoacetate CoA-transferase
2.8.3.18	Succinyl-CoA:acetate CoA-transferase
3.1.1.73	Ferulic acid esterase
3.2.1.8	Endo-1,4-β-xylanase
3.2.1.22	Alpha-galactosidase
3.2.1.37	Xylan 1,4-β-xylosidase
3.2.1.55	Non-reducing end α-L-arabinofuranosidase
3.2.1.136	Glucuronoarabinoxylan endo-1,4-β-xylanase
3.2.1.139	Alpha-glucosiduronase
3.2.1.156	Oligosaccharide reducing-end xylanase
4.1.2.9	Phosphoketolase
5.3.1.5	Xylose isomerase
6.2.1.1	Acetate-CoA ligase
6.2.1.5	Succinyl-CoA synthetase (ADP-forming)
6.2.1.13	Acetate-CoA ligase (ADP-forming)

### Statistical Analysis

Data were analyzed according to the following statistical model:

$$Y_{ijkl}=\mu +\tau_{i}+\upsilon_{j}+\rho_{k}+e_{ijkl}$$

Where *Y_ijkl_* is the observed value for *l*th experimental unit within the *i*th level of dietary treatment of the *j*th block for the *l*th pig in the *k*th replicate; *μ* is the general mean; *τ_i_* is the fixed effect of the *i*th diet (*i* = 1–4); *υ_j_* is the random effect of the *j*th block (*j* = 1–5); *ρ_k_* is the random effect of the *k*th replicate (*k* = 1–3); and *e_ijkl_* is the associated variance as described by the model for *Y_ijkl_* (*l* = 1 through 60).

The top 200 individual OTUs, relative abundance of the 20 most abundant genera, and hidden-state predictions of the 25 microbial enzyme genes were analyzed using a negative binomial distribution in GLIMMIX procedure of SAS (Version 9.4, SAS Inst., Cary, NC), and they were offset by the total library count for a given sample. The MULTTEST procedure of SAS was used to correct *p* values for false discovery rates (*Q* values). For variables with a treatment *Q* < 0.05, the LOG_2_ fold change and respective standard errors, were calculated comparing LF vs. HF, HF vs. HF+XY, and HF vs. HF+AX. Diversity indices were analyzed using PROC MIXED procedure of SAS. Least square means were separated using Fisher’s Least Significant Difference test, and treatment differences were considered significant if *p* < 0.05.

## Results

### Intestinal Microbiota Population Characteristics

A total of 9,280,410 sequences were obtained from all samples after size filtering, quality control, and chimera removal.

#### Cecum

From cecal content samples, a total of 2,707,536 high-quality reads were clustered into 5,889 OTUs and 213 genera, with a median of 41,672 sequences among samples. The five most abundant phyla (Supplementary Figure S1A; 97.5% of all OTUs) present included *Firmicutes* (64.44%), *Bacteroidetes* (27.33%), *Proteobacteria* (2.26%), *Actinobacteria* (2.04%), and *Spirochaetes* (1.40%). When characterized into families (Supplementary Figure S1B), the 20 most abundant families accounted for 96.22% of OTUs, and notably, *Prevotellaceae* (20.90%), *Ruminococcaceae* (19.40%), *Lachnospiraceae* (13.39%), *Clostridiaceae_1* (8.57%), and *Lactobacillaceae* (6.77%) were the five most abundant. Similarly, the 20 most abundant genera accounted for 70.57% of all classified OTUs (Supplementary Figure S1C), and notably *Clostridium_sensu_stricto_1* (8.7%), *Lachnospiraceae_unclassified* (7.84%), *Lactobacillus* (7.65%), *Ruminococcaceae_UCG-005* (5.25%), and *Allopreveotella* (5.07%) rounded out the top five.

A total of 2,551,171 high-quality reads from cecal mucosa samples were clustered into 4,657 OTUs and 234 genera, with a median of 41,672 sequences among samples. The five most abundant phyla among the cecal mucosa-associated microbiota accounted for 95.92% of all OTUs (Supplementary Figure S1D), and encompassed *Firmicutes* (53.08%), *Bacteroidetes* (31.59%), *Epsilonbacteraeota* (4.88%), *Proteobacteria* (4.11%), and *Spirochaetes* (2.27%). When further classified into families, the 20 most abundant accounted for 94.77% of all OTUs (Supplementary Figure S1E), and *Prevotellaceae* (23.44%), *Ruminococcaceae* (14.85%), *Lachnospiraceae* (10.46%), *Streptococcaceae* (6.08%), and *Lactobacillaceae* (5.55%) were the five most abundant. The 20 most abundant genera (Supplementary Figure S1F), comprised 70.22% of all OTUs, and *Allopreveotella* (7.26%), *Streptococcus* (6.08%), *Lactobacillus* (5.54%), *Ruminococcaceae_UCG-005* (5.25%), and *Lachnospiraceae_unclassified* (4.55%) were the five most abundant.

#### Colon

A total of 1,848,354 high-quality reads were obtained from colonic contents and clustered into 2,822 OTUs and 215 genera, with a median of 33,870 sequences among samples. The five most abundant phyla (Supplementary Figure S2A; 96.9% of all OTUs) included *Firmicutes* (66.03%), *Bacteroidetes* (24.42%), *Spirochaetes* (3.51%), *Proteobacteria* (1.50%), and *Euryarchaeota* (1.46%). The 20 most abundant families accounted for 95.6% of OTUs (Supplementary Figure S2B), and notably, *Ruminococcaceae* (18.36%), *Prevotellaceae* (18.04%), *Lachnospiraceae* (16.42%), *Clostridiaceae_1* (11.19%), and *Streptococcaceae* (6.29%) were the five most abundant. Likewise, the 20 most abundant genera comprised 67.4% of all OTUs (Supplementary Figure S2C), and *Clostridium_sensu_stricto_1* (11.05%), *Lachnospiraceae_unclassified* (8.72%), *Streptococcus* (6.29%), *Ruminococcaceae_UCG-005* (5.64%), and *Prevotellaceae_NK3B31_group* (4.53%) composed the five most abundant.

From colonic mucosa samples, a total of 2,173,348 high-quality reads were clustered into 4,516 OTUs and 253 genera, with a median of 37,554 sequences among samples. The five most abundant phyla (Supplementary Figure S2D; 94.2% of all OTUs) present included *Firmicutes* (55.74%), *Bacteroidetes* (27.54%), *Proteobacteria* (4.04%), *Spirochaetes* (3.85%), and *Epsilonbacteraeota* (3.04%). When characterized into families (Supplementary Figure S2E), the 20 most abundant accounted for 91.12% of OTUs, and *Ruminococcaceae* (19.59%), *Prevotellaceae* (16.57%), *Lachnospiraceae* (11.74%), *Streptococcaceae* (7.05%), and *Rikenellaceae* (4.74%) were the five most abundant. With regard to microbial genera, the 20 most abundant accounted for 63.50% of all classified OTUs in the colonic mucosa (Supplementary Figure S2F), and *Streptococcus* (7.05%), *Lachnospiraceae_unclassified* (6.46%), *Ruminococcaceae_UCG-005* (6.22%), *Clostridium_sensu_stricto_1* (4.62%), and *Prevotellaceae_NK3B31_group* (4.12%) were the five most abundant.

#### Alpha-Diversity

**Figure 1:**
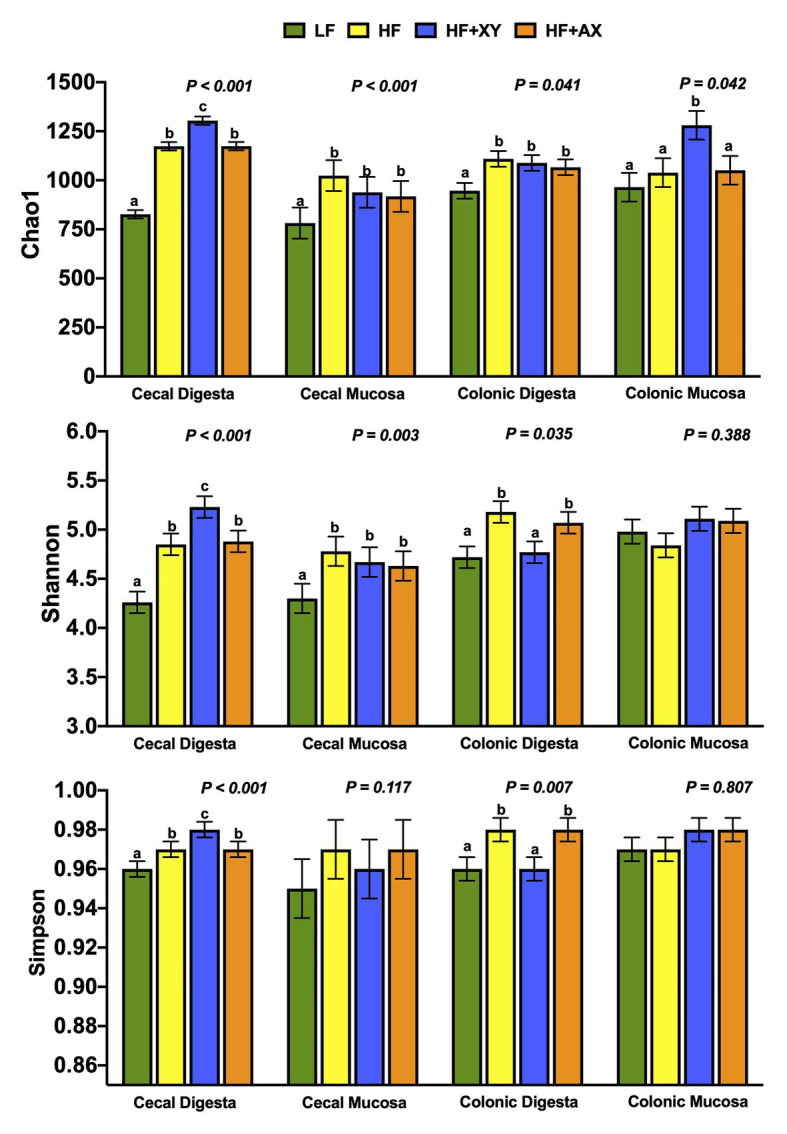
Indices of *α*-diversity of microbial operational taxonomic units (OTUs) among treatments present in the contents and mucosa from the cecum and colon.

Chao1, Shannon, and Simpson indices of diversity, evenness and richness are depicted in Figure 1. For the cecal contents, HF+XY had the greatest Chao1, Shannon, and Simpson indices, with HF and HF+AX performing intermediately among treatments, and LF having the lowest (*p* < 0.001). Compared to LF, HF, HF+XY, and HF+AX had greater Chao1 and Shannon indices in the cecal mucosa (*p* < 0.05), but did not differ for the Simpson index (*p* = 0.117). Similarly, for colonic contents HF, HF+XY, and HF+AX had a greater Chao1 index than LF (*p* = 0.041). For the Shannon and Simpson indices, HF and HF+AX were greater than LF and HF+XY (*p* < 0.05). Shannon and Simpson indices did not differ among treatments in the colonic mucosa, but HF+XY had the greatest Chao1 index (*p* = 0.042).

### Dietary Treatment Modulation of Cecal and Colonic Microbiota

**Figure 2:**
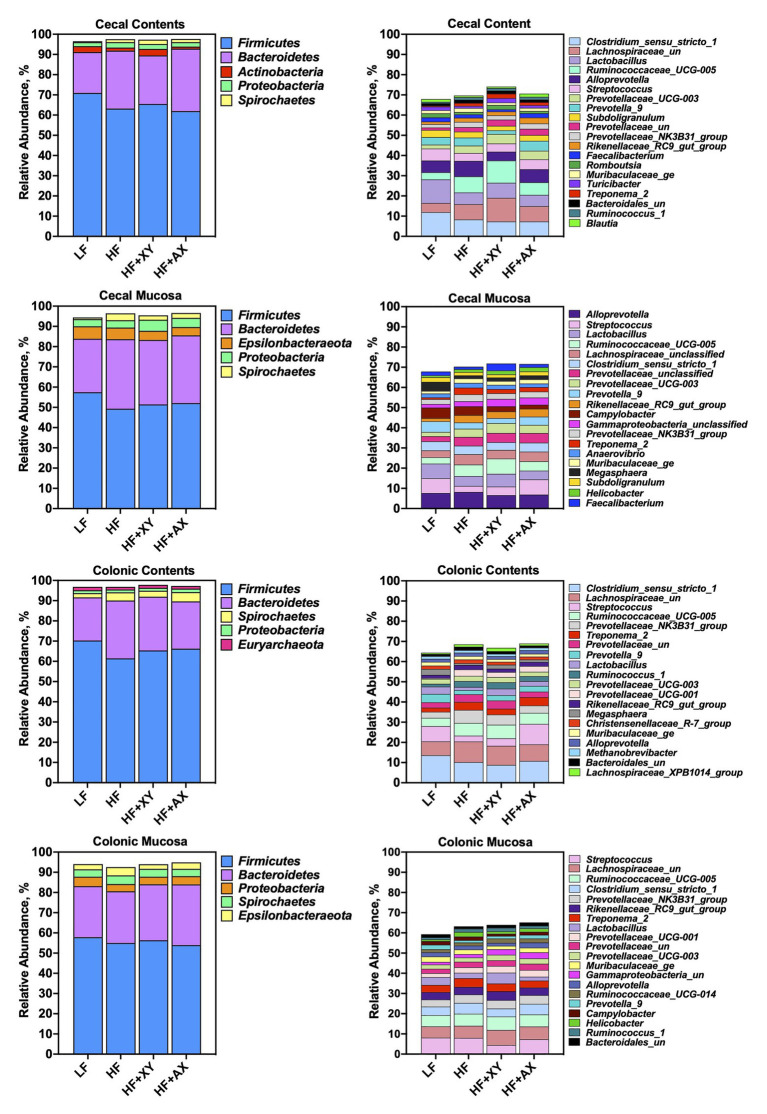
The relative abundance of the five most abundant phyla and 20 most abundant genera among treatments found in the contents and mucosa from the cecum and colon.

Figure 2 depicts the relative abundance of the five most abundant phyla and 20 most abundant genera among treatments within each location. The five most abundant phyla accounted for 96.5, 97.5, 97.3, and 97.7% of all characterized OTUs in cecal contents for LF, HF, HF+XY, and HF+AX, respectively. Whereas, the 20 most abundant genera among treatments accounted for 67.9, 69.7, 74.1, and 70.6% of all OTUs present in cecal contents for LF, HF, HF+XY, and HF+AX, respectively. In the cecal mucosa, the five most abundant phyla comprised 94.4, 96.4, 95.4, and 96.6% of all OTUs characterized in LF, HF, HF+XY, and HF+AX, respectively. Likewise, the 20 most abundant genera among microbiota characterized from cecal mucosa accounted for 67.8, 70.2, 71.8, and 71.6% of the OTUs characterized in LF, HF, HF+XY, and HF+AX, respectively.

In the colonic contents, the five most abundant phyla comprised 96.8, 96.8, 97.8, and 97.3% of all OTUs present in LF, HF, HF+XY, and HF+AX, respectively. Whereas, in the colonic mucosa, the five most abundant phyla accounted for 94, 92.6, 94, and 94.9% of the OTUs is present in LF, HF, HF+XY, and HF+AX, respectively. The 20 most abundant genera in the colonic contents composed 64.4, 68.5, 66.8, and 68.9% of the OTUs found in LF, HF, HF+XY, and HF+AX, respectively. Within the colonic mucosa, the 20 most abundant genera comprised 59.3, 63.2, 63.9, and 65.2% of OTUs found in in LF, HF, HF+XY, and HF+AX, respectively.

#### Treatment Modulation of Microbial Genera in the Cecum

**Figure 3:**
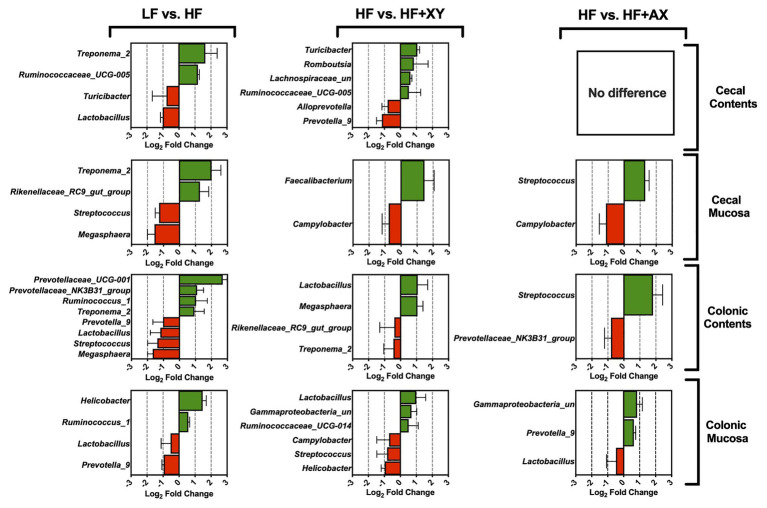
The Log_2_ fold change difference between LF vs. HF, HF vs. HF+XY, and HF vs. HF+AX for the 20 most abundant microbial genera in the contents and mucosa from the cecum and colon with a value of *Q* < 0.05.

Relative to LF, HF increased *Treponema_2* and *Ruminococcaceae_UCG-005*, and reduced *Turicibacter* and *Lactobacillus* in cecal contents (Figure 3; *Q* < 0.05). In the cecal mucosa, relative to LF, HF had a greater proportion of *Treponema_2* and *Rikenellaceae_RC9_gut_group*, and *Megasphaera* and *Streptococcus* were less abundant (Figure 3; *Q* < 0.05). The addition of xylanase to HF increased the presence of *Turicibacter*, *Romboutsia*, *Lachnospiraceae_unclassified*, and *Ruminococcaceae_UCG-005*, but decreased the prevalence of *Prevotella_9* and *Alloprevotella*, in the cecal contents (*Q* < 0.05). Relative to HF, HF+XY increased *Faecalibacterium* in the cecal mucosa, and decreased the prevalence of *Campylobacter* (*Q* < 0.05). The 20 most abundant genera did not differ between HF and HF+AX in the cecal contents (*Q* > 0.10), but HF+AX did increase the abundance of *Streptococcus* and decreased the prevalence of *Campylobacter* (*Q* < 0.05).

#### Treatment Modulation of Microbial Genera in the Colon

In the colonic contents, relative to LF, HF increased the prevalence of *Prevotellaceae_UCG-001*, *Prevotellaceae_NK3B31_group*, *Ruminococcus_1*, and *Treponema_2*, but decreased the abundance of *Prevotella_9*, *Lactobacillus*, *Streptococcus*, and *Megasphaera* (Figure 3; *Q* < 0.05). Similarly, compared to LF, HF decreased the abundance of *Prevotella_9* and *Lactobacillus* in the colonic mucosa, but increased *Helicobacter* and *Ruminococcus_1* (Figure 3; *Q* < 0.05). Contrarily in the colonic contents, when compared to HF, HF+XY increased the abundance of *Megasphaera* and *Lactobacillus* but decreased the prevalence of *Rikenellaceae_RC9_gut_group* and *Treponema_2* (*Q* < 0.05). Moreover, the addition of xylanase to HF increased the abundance of *Lactobacillus*, *Gammaproteobacteria_unclassified*, and *Ruminococcaceae_UCG-014* in the colonic mucosa, but decreased the abundance of *Campylobacter*, *Streptococcus*, and *Helicobacter* (*Q* < 0.05). The addition of AXOS to HF increased *Streptococcus* and decreased *Prevotellaceae_NK3B31_group* in the colonic contents (*Q* < 0.05). Within the colonic mucosa, compared with the HF, HF+AX increased the abundance of *Prevotella_9* and *Gammaproteobacteria_unclassified*, but reduced the abundance of *Lactobacillus* (*Q* < 0.05).

#### Treatment Modulation of the 200 Most Abundant OTUs in the Cecal Contents

**Figure 4:**
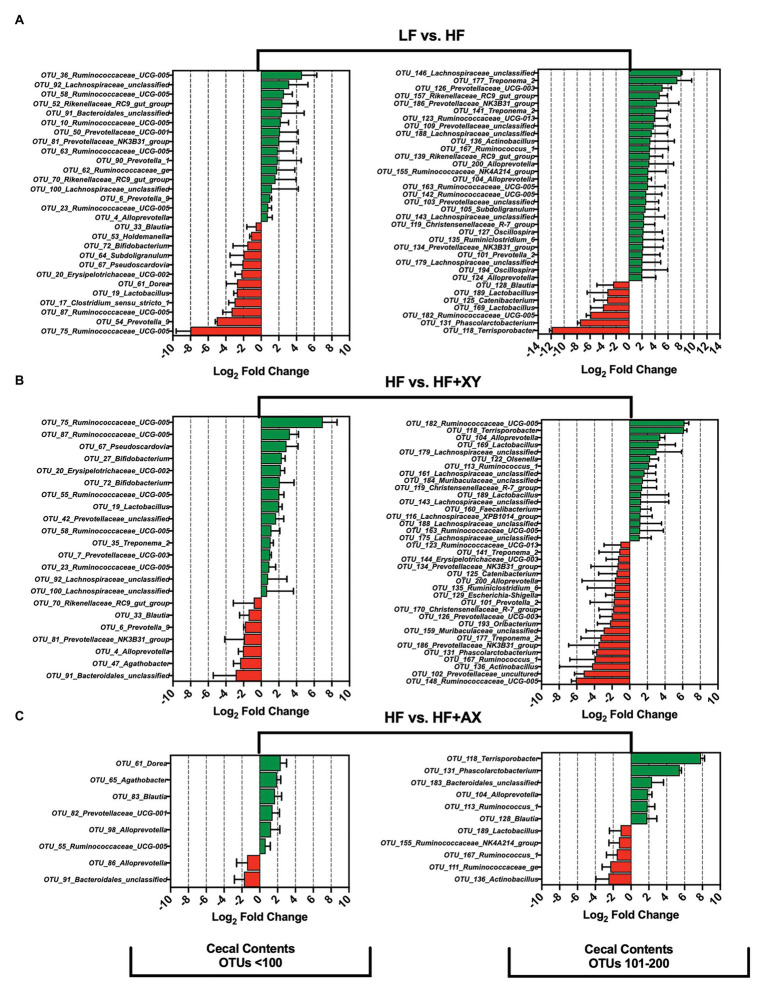
The Log_2_ fold change difference between **(A)** LF vs. HF, **(B)** HF vs. HF+XY, and **(C)** HF vs. HF+AX or OTUs with a contrast value of *Q* < 0.05 from the 200 most abundant OTUs among treatments present in the cecal contents.

Compared to LF, HF significantly increased the prevalence of 44, and reduced 19, of the 200 most prevalent OTUs characterized from cecal contents (Figure 4A). Notably, among the 100 most abundant OTUs in the cecal contents, HF increased the prevalence of five OTUs from the genus *Ruminococcaceae_UCG-005* (OTUs #10, 23, 36, 58, and 63), two unclassified OTUs associated with the family *Lachnospiraceae* (OTUs #92 and 100), two OTUs from the genus *Rikenellaceae_RC9_gut_group* (OTUs #52 and 70), and five OTUs from the family *Prevotellaceae* (OTUs: *OTU_4_Alloprevotella*, *OTU_6_Prevotella_9*, *OTU_50_Prevotellaceae_UCG-001*, *OTU_81_Prevotellaceae_NK3B31_group*, and *OTU_90_Prevotella_1*). Interestingly, among the 100 most abundant OTUs in the cecal contents, HF significantly decreased the prevalence of *OTU_17_Clostridium_sensu_stricto_1*, *OTU_19_Lactobacillus*, *OTU_33_Blautia*, *OTU_53_Holdemanella*, *OTU_64_Subdoligranulum*, *OTU_67_Pseudoscardovia*, and *OTU_72_Bifidobacterium*. For OTUs 101 through 200 in the cecal contents, when compared to LF, HF increased the prevalence of four unclassified OTUs from the family *Lachnospiraceae* (OTUs #143, 146, 179, and 188), two OTUs from the genus *Rikenellaceae_RC9_gut_group* (OTUs #139 and 157), two OTUs from the genus *Treponema_2* (OTUs #141 and 177), five OTUs from the family *Ruminococcaceae* (OTUs #123, 142, 155, 163, and 167), and two from the genus *Oscillospira* (OTUs #127 and 194). However, relative to LF, HF decreased the abundance of *OTU_118_Terrisporobacter*, *OTU_131_Phascolarctobacterium*, *OTU_182_Ruminococcaceae_UCG-005*, *OTU_169_Lactobacillus*, *OTU_125_Catenibacterium*, *OTU_189_Lactobacillus*, and *OTU_128_Blautia*, in the cecal contents. The addition of xylanase to HF significantly increased 32, and decreased 27, of the 200 most prevalent OTUs characterized from cecal contents (Figure 4B). Notably, HF+XY increased the prevalence of seven unclassified OTUs from the family *Lachnospiraceae* (OTUs #92, 100, 143, 161, 175, 179, and 188), seven OTUs from the genus *Ruminococcaceae_UCG-005* (OTUs #23, 55, 58, 75, 87, 163, and 182), three OTUs from the genus *Lactobacillus* (OTUs #19, 169, and 189), two from the *Bifidobacterium* genus (OTUs # 27and 72), and increased the abundance of *OTU_160_Faecalibacterium*. Moreover, relative to HF, HF+XY decreased the prevalence of nine OTUs from the family *Prevotellaceae* (OTUs #4, 6, 81, 101, 102, 126, 134, 186, and 200) and decreased the abundance of *OTU_47_Agathobacter* and *OTU_129_Escherichia-Shigella* in the cecal contents. Compared to HF, HF+AX increased the abundance of 12, and decreased seven, of the 200 most prevalent OTUs characterized from cecal contents (Figure 4C).

#### Treatment Modulation of the 200 Most Abundant OTUs in the Cecal Mucosa

**Figure 5:**
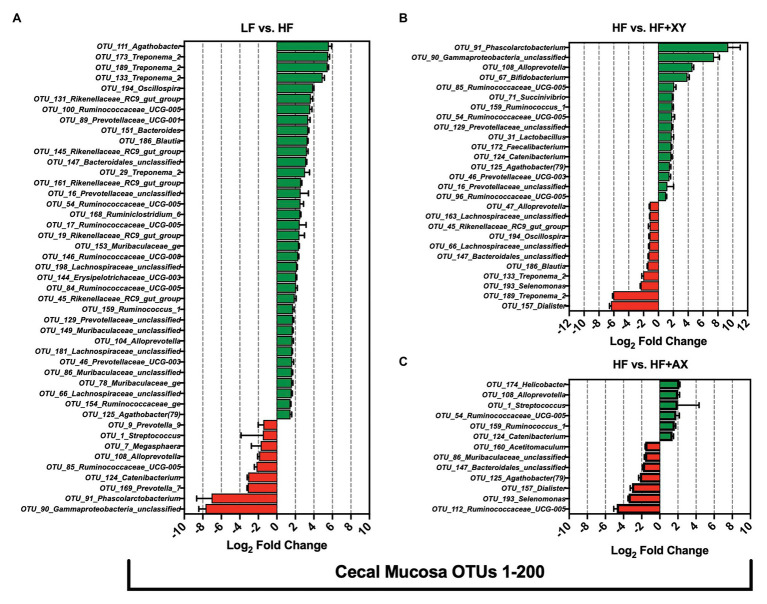
The Log_2_ fold change difference between **(A)** LF vs. HF, **(B)** HF vs. HF+XY, and **(C)** HF vs. HF+AX or OTUs with a contrast value of *Q* < 0.05 from the 200 most abundant OTUs among treatments present in the cecal mucosa.

Compared to LF, HF significantly increased 36, and decreased nine, of the 200 most abundant OTUs characterized in the cecal mucosa (Figure 5A). In the cecal mucosa, relative to LF, HF increased the abundance of seven OTUs from the family *Ruminococcaceae* (OTUs #17, 54, 84, 100, 146, 154, and 159), five OTUs from the *Rikenellaceae_RC9_gut_group* genus (OTUs #19,45,131,145, and 161), five OTUs from the *Prevotellaceae* family (OTUs #16, 46, 89,104, 129), four OTUs from the *Muribaculaceae* family (OTUs #78,86,149, and 153), four OTUs associated with the *Treponema*_2 genus (OTUs #29, 133, 173,and 189), and three unclassified OTUs from the *Lachnospiraceae* family (OTUs #66, 181, and 198). Interestingly, relative to LF, HF decreased the abundance of *OTU_1_Streptococcus*, *OTU_7_Megasphaera*, and *OTU_9_Prevotella_9*, in the cecal mucosa. Relative to HF, HF+XY significantly increased 16, and decreased 11, of the 200 most abundant OTUs characterized in the cecal mucosa (Figure 5B). Compared to HF, in the cecal mucosa, HF+XY increased the prevalence of three OTUs from the *Ruminococcaceae_UCG-005* genus (OTUs #54, 85, and 96), and three OTUs from the *Prevotellaceae* family (OTUs #16, 46, and 129). Interestingly, HF+XY also increased the prevalence of *OTU_31_Lactobacillus*, *OTU_67_Bifidobacterium*, *OTU_71_Succinivibrio*, *OTU_124_Catenibacterium*, and *OTU_172_Faecalibacterium* in the cecal mucosa. The addition of AXOS to HF significantly increased five, and decreased seven of the 200 most abundant OTUs (Figure 5C). Notably, HF+AX had a greater abundance of *OTU_1_Streptococcus* and *OTU_174_Helicobacter*, and decreased prevalence of *OTU_193_Selenomonas* and *OTU_160_Acetitomaculum*.

#### Treatment Modulation of the 200 Most Abundant OTUs in the Colonic Contents

**Figure 6:**
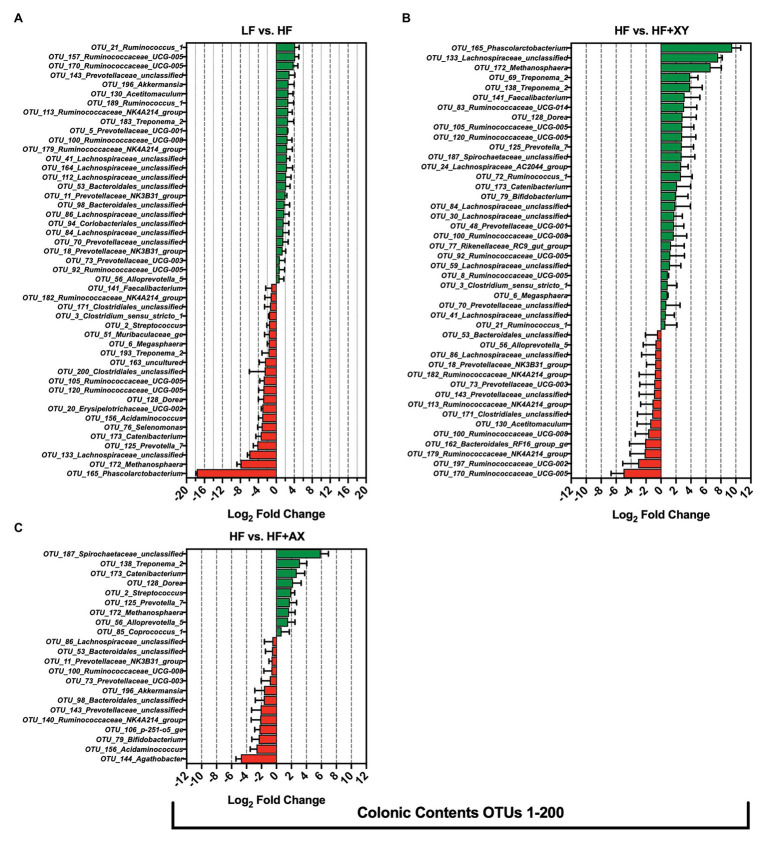
The Log_2_ fold change difference between **(A)** LF vs. HF, **(B)** HF vs. HF+XY, and **(C)** HF vs. HF+AX or OTUs with a contrast value of *Q* < 0.05 from the 200 most abundant OTUs among treatments present in the colonic contents.

Compared to LF, HF significantly increased 26, and decreased 21, of the 200 most abundant OTUs characterized in the colonic contents (Figure 6A). Relative to LF, HF increased the abundance of eight OTUs from the family *Ruminococcaceae* (OTUs #21, 92, 100, 113, 157, 170, 179, and 189), seven OTUs from the *Prevotellaceae* family (OTUs #5, 11, 18, 56, 70, 73, and 143), five unclassified OTUs from the *Lachnospiraceae* family (OTUs #41, 84, 86, 112, and 164), and increased the abundance of *OTU_130_Acetitomaculum* and *OTU_196_Akkermansia*. Interestingly, relative to LF, HF decreased the abundance of *OTU_2_Streptococcus*, *OTU_6_Megasphaera*, *OTU_76_Selenomonas*, *OTU_128_Dorea*, *OTU_141_Faecalibacterium*, *OTU_165_Phascolarctobacterium*, and *OTU_172_Methanosphaera* in the colonic contents. The addition of xylanase to HF significantly increased 29, and decreased 15, of the 200 most abundant OTUs characterized in the colonic contents (Figure 6B). Compared to HF, HF+XY increased the abundance of eight OTUs from the family *Ruminococcaceae* (OTUs #8, 21, 72, 83, 92, 100, 105, and 120), and five OTUs from the *Lachnospiraceae* family (OTUs #24, 30, 41, 59, 84, and 133) in the colonic contents. Moreover, compared to HF, HF+XY increased the abundance of *OTU_6_Megasphaera*, *OTU_79_Bifidobacterium*, *OTU_128_Dorea*, *OTU_141_Faecalibacterium*, *OTU_165_Phascolarctobacterium*, *OTU_172_Methanosphaera*, and *OTU_173_Catenibacterium* in the colonic contents. Compared to HF, HF+AX increased the abundance of nine, and decreased 12, of the 200 most prevalent OTUs characterized from colonic contents (Figure 6C). Notably, the addition of AXOS to HF increased the abundance of *OTU_2_Streptococcus*, *OTU_56_Alloprevotella_5*, *OTU_85_Coprococcus_1*, *OTU_125_Prevotella_7*, *OTU_128_Dorea*, *OTU_138_Treponema_2*, *OTU_172_Methanosphaera*, *OTU_173_Catenibacterium*, and *OTU_187_Spirochaetaceae_unclassified* in the colonic contents. However, HF+AX had decreased prevalence of *OTU_79_Bifidobacterium*, *OTU_140_Ruminococcaceae_NK4A214_group*, *OTU_143_Prevotellaceae_unclassified*, *OTU_144_Agathobacter*, *OTU_156_Acidaminococcus*, and *OTU_196_Akkermansia*, compared to HF in colonic contents.

#### Treatment Modulation of the 200 Most Abundant OTUs in the Colonic Mucosa

**Figure 7:**
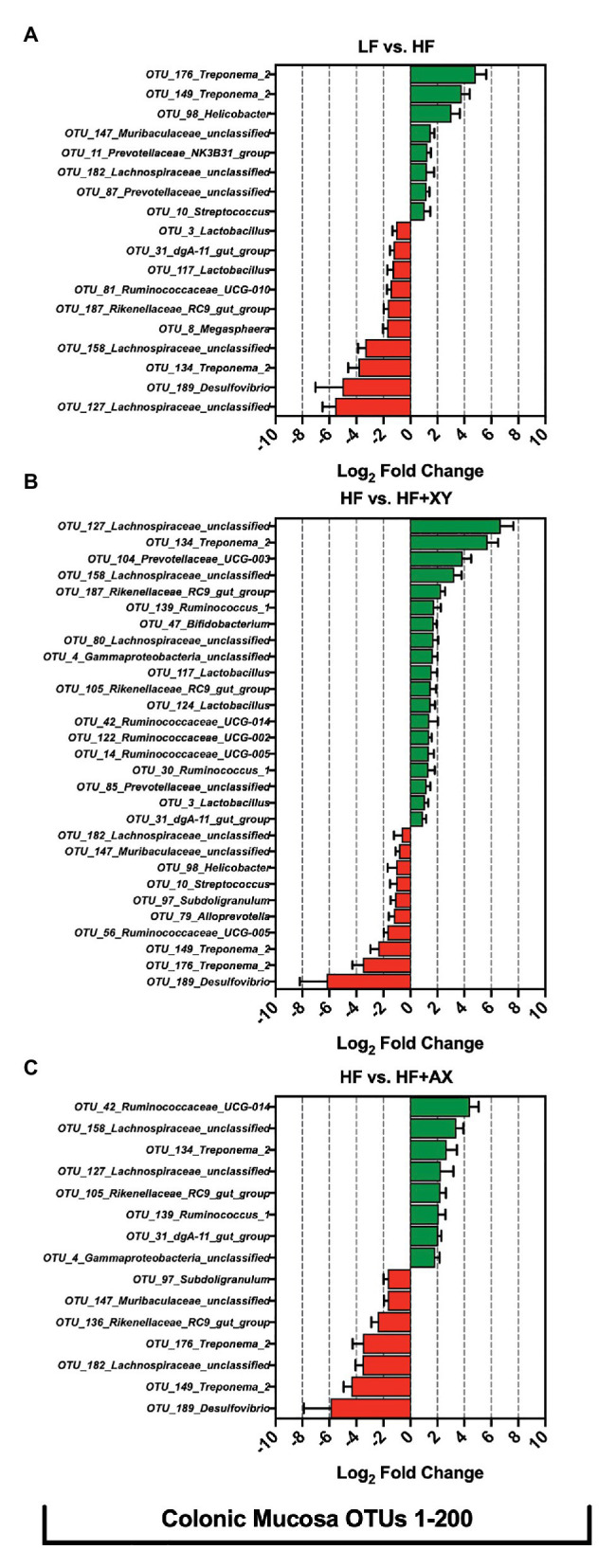
The Log_2_ fold change difference between **(A)** LF vs. HF, **(B)** HF vs. HF+XY, and **(C)** HF vs. HF+AX for OTUs with a contrast value of *Q* < 0.05 from the 200 most abundant OTUs among treatments present in the colonic mucosa.

Relative to LF, HF significantly increased eight of the 200 most abundant OTUs characterized from colonic mucosa (Figure 7A): *OTU_176_Treponema_2*, *OTU_149_Treponema_2*, *OTU_98_Helicobacter*, *OTU_147_Muribaculaceae_unclassified*, *OTU_11_Prevotellaceae_NK3B31_group*, *OTU_182_Lachnospiraceae_unclassified*, *OTU_87_Prevotellaceae_unclassified*, and *OTU_10_Streptococcus*. Moreover, compared to LF, HF had decreased abundance of 10 of the 200 most abundant OTUs characterized from colonic mucosa: *OTU_127_Lachnospiraceae_unclassified*, *OTU_189_Desulfovibrio*, *OTU_134_Treponema_2*, *OTU_158_Lachnospiraceae_unclassified*, *OTU_8_Megasphaera*, *OTU_187_Rikenellaceae_RC9_gut_group*, *OTU_81_Ruminococcaceae_UCG-010*, *OTU_117_Lactobacillus*, *OTU_31_dgA-11_gut_group*, and *OTU_3_Lactobacillus*. Relative to HF, HF+XY significantly increased 19, and decreased 10, of the 200 most abundant OTUs characterized from colonic mucosa (Figure 7B). Compared to HF, in the colonic mucosa, HF+XY increased the prevalence of five OTUs from the *Ruminococcaceae* family (OTUs #14, 30, 42, 122, 134, and 139), three OTUs from the *Lachnospiraceae* family (OTUs #80, 127, and 158), three OTUs from the *Lactobacillus* genus (OTUs #3, 117, and 124), two OTUs from the *Prevotellaceae* family (OTUs #85 and 104), and two OTUs from the *Rikenellaceae_RC9_gut_group* (OTUs #105 and 187). Moreover, HF+XY had a greater abundance of *OTU_47_Bifidobacterium*, *OTU_31_dgA-11_gut_group*, and *OTU_4_Gammaproteobacteria_unclassified* in the colonic mucosa. Contrarily, the addition of xylanase to HF reduced the abundance of *OTU_189_Desulfovibrio*, *OTU_176_Treponema_2*, *OTU_149_Treponema_2*, *OTU_56_Ruminococcaceae_UCG-005*, *OTU_79_Alloprevotella*, *OTU_97_Subdoligranulum*, *OTU_10_Streptococcus*, *OTU_98_Helicobacter*, *OTU_147_Muribaculaceae_unclassified*, and *OTU_182_Lachnospiraceae_unclassified* in the colonic mucosa. Relative to HF, HF+AX increased the abundance of eight OTUs in the colonic mucosa (Figure 7C): *OTU_42_Ruminococcaceae_UCG-014*, *OTU_158_Lachnospiraceae_unclassified*, *OTU_134_Treponema_2*, *OTU_127_Lachnospiraceae_unclassified*, *OTU_105_Rikenellaceae_RC9_gut_group*, *OTU_139_Ruminococcus_1*, *OTU_31_dgA-11_gut_group*, and *OTU_4_Gammaproteobacteria_unclassified*. Moreover, compared to HF, HF+AX had reduced abundance of seven OTUs in the colonic mucosa (Figure 7C): *OTU_189_Desulfovibrio*, *OTU_149_Treponema_2*, *OTU_182_Lachnospiraceae_unclassified*, *OTU_176_Treponema_2*, *OTU_136_Rikenellaceae_RC9_gut_group*, *OTU_147_Muribaculaceae_unclassified*, and *OTU_97_Subdoligranulum*.

### PICRUSt2 Targeted Gene Characterizations

**Figure 8:**
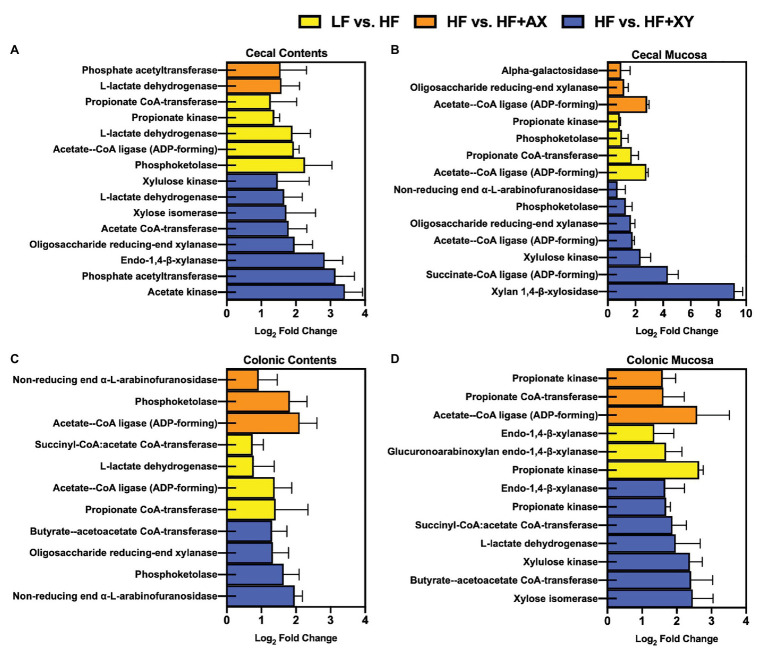
The Log_2_ fold change between LF vs. HF, HF vs. HF+XY, and HF vs. HF+AX for the PICRUSt2 significantly different predicted gene counts for enzymes associated with bacterial arabinoxylan degradation, xylose metabolism, or SCFA metabolism found in the **(A)** cecal contents, **(B)** cecal mucosa, **(C)** colonic contents, and **(D)** colonic mucosa.

Relative to LF, HF increased the abundance of predicted gene counts for propionate kinase, L-lactate dehydrogenase, acetate-CoA ligase [adenosine diphosphate (ADP)-forming], and phosphoketolase in the cecal contents (Figure 8A; *Q* < 0.05). The addition of xylanase to HF increased predicted gene counts for acetate kinase, phosphate acetyltransferase, endo-1,4-beta-xylanase, acetate CoA-transferase, xylose isomerase, L-lactate dehydrogenase, and xylulose kinase (*Q* < 0.05). Compared to HF, HF+AX increased the abundance of predicted gene counts for phosphate acetyltransferase and L-lactate dehydrogenase (*Q* < 0.05).

In the cecal mucosa, relative to LF, HF increased predicted gene counts for acetate-CoA ligase (ADP-forming), propionate CoA-transferase, phosphoketolase, and propionate kinase (Figure 8B; *Q* < 0.05). Compared to HF, HF+XY increased xylan 1,4-β-xylosidase, succinate-CoA ligase (ADP-forming), xylulose kinase, acetate-CoA ligase (ADP-forming), oligosaccharide reducing-end xylanase, phosphoketolase, and non-reducing end alpha-L-arabinofuranosidase predicted gene counts in the cecal mucosa (*Q* < 0.05). The addition of AXOS to HF increased acetate-CoA ligase (ADP-forming), oligosaccharide reducing-end xylanase, and alpha-galactosidase predicted gene counts in the cecal mucosa.

In the colonic contents, relative to LF, HF increased predicted gene counts for propionate CoA-transferase, acetate-CoA ligase (ADP-forming), L-lactate dehydrogenase, and succinyl-CoA:acetate CoA-transferase (Figure 8C; *Q* < 0.05). The addition of xylanase to HF increased predicted gene counts for non-reducing end alpha-L-arabinofuranosidase, phosphoketolase, oligosaccharide reducing-end xylanase, and butyrate-acetoacetate CoA-transferase in the colonic contents (*Q* < 0.05). Likewise, compared to HF, HF+AX pregulated acetate-CoA ligase (ADP-forming), phosphoketolase, and non-reducing end alpha-L-arabinofuranosidase predicted gene counts in colonic content (*Q* < 0.05).

In the colonic mucosa, compared to LF, HF has a greater abundance of predicted gene counts for propionate kinase, glucuronoarabinoxylan endo-1,4-beta-xylanase, and endo-1,4-beta-xylanase (Figure 8D; *Q* < 0.05). Relative to HF, HF+XY increased xylose isomerase, butyrate-acetoacetate CoA-transferase, xylulose kinase, L-lactate dehydrogenase, succinyl-CoA:acetate CoA-transferase, propionate kinase, and endo-1,4-beta-xylanase predicted gene counts in the colonic mucosa (*Q* < 0.05). Compared to HF, HF+AX increased acetate-CoA ligase (ADP-forming), propionate CoA-transferase, and propionate kinase predicted gene counts in the colonic mucosa (*Q* < 0.05).

## Discussion

In swine nutrition, there has been a renewed interest in the beneficial aspects of DF, and particularly, its ability to modulate microbiota and gut health (Jha et al., 2019). However, the DF found in swine diets is often insoluble and poorly fermented by the hindgut microbiota, and often stems from corn and corn co-products (Gutierrez et al., 2013). Arabinoxylans encompass nearly half of the DF found in corn and several corn co-products (Jaworski et al., 2015). Corn-based arabinoxylans are poorly fermented due to the degree of interaction with other plant components, arabinose substitutions, abundant phenolic cross linkages, and lignification (Bach Knudsen, 2014). One strategy that may improve the fermentability of corn-based arabinoxylans is to include xylanase into the diet. Xylanase hydrolyzes the β-(1-4) glycosidic bonds of arabinoxylans, and potentially releases AXOS, and more fragmented and depolymerized arabinoxylans (Dodd and Cann, 2009; Pedersen et al., 2015). Thus, it is logical that xylanase supplementation may improve DF fermentation, as the greater the degree and rate of depolymerization of DF, the faster and more efficiently it can be fermented by microbiota (Varel and Yen, 1997). Moreover, there is increasing evidence in poultry that AXOS can invoke a stimbiotic MOA that modulates microbial communities to support fiber degradation beyond what is feasible from fermentation of AXOS alone (González-Ortiz et al., 2019). The aim of this research was to characterize the modulation of the hindgut microbiota due to increased corn-based fiber, xylanase, or directly supplemented AXOS in pigs fed corn-based diets for 46 days. It was hypothesized that the addition of xylanase or AXOS to a diet high in DF would modulate intestinal microbial communities to support greater fiber degradation through a stimbiotic MOA.

The overall composition of microbial phyla and families observed in the cecum are comparable to what has been defined as “core microbiota” in pigs through a meta-analysis conducted by Holman et al. (2017). This is also the case for the overall composition of microbial phyla observed in the colon, with the exception of the increased prevalence of *Spirochaetes*. However, *Spirochaetes* have been observed at a similar relative abundance in the colon of pigs raised under commercial conditions (De Rodas et al., 2018). Similar to what Holman et al. (2017) observed, *Bacteroidetes* has an increased prevalence in the mucosa, and *Firmicutes* were more dominant in the contents of the large intestine. At the family level, among all locations, *Prevotellaceae*, *Ruminococcaceae*, and *Lachnospiraceae* were the three most dominant families present, but when further classified into genera there were unique differences among which genera prevailed at each location. Moreover, the microbial genera characterized herein are typically present in the cecum and colon of commercially raised pigs (De Rodas et al., 2018; Wang et al., 2019). Intriguingly, within this study, there appears to be two discriminant shifts in microbial composition among dietary treatments: those due to increased DF (LF vs. HF), and those due to the addition of xylanase to HF (HF vs. HF+XY).

Relative to a typical corn-soybean meal basal diet (LF), HF increased measures of *α*-diversity and microbial richness in the cecal contents and mucosa, and colonic contents. These differences in microbial diversity and the modulation of cecal and colonic microbiota by HF, are likely a result of increased DF in the ileal digesta entering the large intestine. The HF treatment had a 30% addition of corn bran without solubles, which increased the NDF content of the diet by nearly 3-fold compared to LF. Moreover, LF had increased ileal digestibility of both DM and NDF in a related study (Petry et al., 2019b), and thus, would have less NDF entering the hindgut to be utilized as a substrate for microbiota. It has been established that diets enriched with DF promote microbial diversity in the large intestine of humans (Sonnenburg et al., 2016; Makki et al., 2018). Moreover, the increase in microbial diversity by HF is in agreement with Liu et al. (2018) who observed increased in the Shannon index in fecal microbiota from nursery pigs fed corn bran at an inclusion rate of 5%.

Within the cecal contents, HF increased the abundance of *Ruminococcaceae_UCG-005*, and in general, microbes in the *Ruminococcaceae* family are known to degrade and metabolize complex plant carbohydrates (Biddle et al., 2013; Chatellard et al., 2016). Moreover, HF increased the prevalence of *Treponema_2* in the cecal mucosa and contents. While little is known about *Treponema_2* in the swine microbiota, in ruminants, *Treponema* is known to increase in concert with cellulolytic bacteria such as *Ruminococcaceae_UCG-005* (Karri et al., 2016; Xie et al., 2018). This is further supported by the drastic increase in *Treponema_2* by HF in the colonic mucosa, and the modulation of *Prevotellaceae*. In contrast, relative to LF, HF decreased *Turicibacter* and *Lactobacillus* in the cecal contents, and *Megasphaera* and *Streptococcus* in the mucosa. *Turicibacter*, although there is limited information about this genus, has been found to increase in humans who consume low fiber diets (Gomez-Arango et al., 2018) and is positively correlated with increased body weight in pigs (Wang et al., 2019). Indeed, LF is lower in DF than HF, and pigs fed LF had increased BW in a related study (Petry et al., 2020a). The reduction is *Megasphaera* by HF, or inversely, a greater abundance of *Megasphaera* in LF, is in agreement with research that suggest pigs with greater feed efficiency, such as LF, have a greater abundance of *Megasphaera* (Tan et al., 2018; Petry et al., 2020a). In the colonic contents and mucosa, HF increased the abundance of *Ruminococcus_1*, and at the OTU level, *Ruminococcus_1* was increased in the cecal contents and mucosa. The increased abundance of *Ruminococcus_1* could be a result of increased mucin production as species within the *Ruminococcus* genus are prolific degraders of the polyglycans found in mucins (Crost et al., 2018). Furthermore, the abrasiveness of insoluble DF can increase mucin production in the gastrointestinal tract (Montagne et al., 2004; Agyekum and Nyachoti, 2017).

Across all locations, HF increased the abundance of various OTUs from the *Lachnospiraceae*, *Ruminococcaceae*, and *Prevotellaceae* families. Many species within these families have the potential to ferment diverse and complex polysaccharides (Biddle et al., 2013; Meehan and Beiko, 2014; Chen et al., 2017). Moreover, the increase in predicted gene counts for enzymes associated with propionate production by HF support the degradation of DF by *Prevotellaceae* (Chen et al., 2017). However, in Petry et al. (2019b, 2020b), compared to LF, HF had reduced SCFA concentrations and NDF digestibility in cecal and colonic contents. Interestingly, a study by Quan et al. (2018) found pigs with poor feed efficiency had a greater abundance of *Lachnospiraceae*, *Ruminococcaceae*, and *Prevotellaceae* families in the cecum and colon compared to more feed efficient pigs fed the same corn-soybean meal-based diet. It is plausible that these families are less efficient at fermenting DF, compared to the microbial communities that were more abundant in LF.

Supplementing xylanase to HF increased all measures of *α*-diversity in the cecal content, and HF+XY had the greatest Chao1 index among treatments in the colonic mucosa. Including a carbohydrase blend that contained xylanase in wheat bran, and soybean hull-based swine diets increased the Chao1 and Shannon indices of ileal digesta and feces (Zhang et al., 2018a). However, Zhang et al. (2018b) observed no differences in the Shannon and Simpson indices of cecal contents from pigs fed a corn distiller’s dried grains with solubles (DDGS)-based diet supplemented with xylanase with a similar dietary adaptation time. These opposing results are potentially due to the recalcitrant nature of corn DDGS to xylanase hydrolysis (Abelilla and Stein, 2019), whereas, corn-bran appears to be more susceptible to xylanase (Petry et al., 2020a).

Directly supplementing AXOS did not alter *α*-diversity measurements in the colonic contents, with the exception of greater Simpson and Chao1 indices than LF and HF+XY, but not HF. Moreover, HF+AX did not modulate microbial communities to the same degree, or in a similar manner, to HF+XY. This is counterintuitive to what has been observed in poultry, and the prebiotic-like effect of AXOS supplementation in humans (Grootaert et al., 2007; Morgan et al., 2019; Bautil et al., 2020). It is plausible that AXOS produced *in situ* by xylanase differs in composition and degrees of polymerization due to the intrinsic complexities of corn-based arabinoxylans. Moreover, pigs, compared to poultry, have a longer intestine relative to their body weight (Moran, 1982). Thus, it is reasonable to speculate that the AXOS in HF+AX could have been fermented in the small intestine, and unable to modulate hindgut microbiota. Xylanase also has the potential to degrade arabinoxylans across the gastrointestinal tract, and continually produce soluble AXOS throughout the large intestine.

The most recently proposed MOA of xylanase suggests the *in situ* produced AXOS stimulates gastrointestinal microbiota to digest DF to a greater degree than the fermentation of AXOS alone (Bedford, 2018). This was later coined as a “stimbiotic” or “stimbiotic mechanism” by González-Ortiz et al. (2019). Compared to HF, HF+XY increased predicted genes of microbial enzymes associated with pentose metabolism and arabinoxylan degradation in cecal contents, cecal mucosa, and colonic mucosa (Figure 8). These findings would suggest xylanase does indeed stimulate a fiber-fermenting microbiome in the large intestine of the pig. Moreover, within cecal and colonic contents, HF+XY increased the abundance of several OTUs within the *Lachnospiraceae* and *Ruminococcaceae* families to a greater degree than their modulation by HF alone. Genomic investigation into the metabolic capacities of *Lachnospiraceae* and *Ruminococcaceae* suggest these microbial communities are able to metabolize xylose and produce microbial carbohydrases (Biddle et al., 2013). These data, in conjunction with the improved cecal digestibility of NDF observed by Petry et al. (2019b), further support the premise that xylanase elicits a stimbiotic MOA. Likewise, the upregulation of xylan 1,4-β-xylosidase and alpha-L-arabinofuranosidase predicted gene counts within the cecal mucosa also suggest liberated AXOS are being fermented within the cecum. This is further supported by the findings of Petry et al. (2020b), who observed HF+XY increased both the absolute concentration and molar proportion of acetate in the cecum.

Interestingly, xylanase across locations increased *Lactobacillus*, *Bifidobacterium*, and *Faecalibacterium* at either the genus or OTU level. The increased abundance of *Lactobacillus* and *Bifidobacterium* by xylanase has also been observed in poultry (Vahjen et al., 1998; González-Ortiz et al., 2020). The increased prevalence of these microbial communities implies HF+XY promoted beneficial cross feeding that would favor acetate and butyrate production in the large intestine (Zeybek et al., 2020). Several species within the *Lactobacillus* genus have the capacity to utilize more polymerized and complex AXOS, and this often results in the production of small AXOS, lactate, and acetate (Immerzeel et al., 2014; McLaughlin et al., 2015). *Bifidobacterium* are likely feeding on the short AXOS liberated by exogenous xylanase, *Lactobacillus*, and other fibrolytic microbiota (Rivière et al., 2014). The increase in *Lactobacillus* and *Bifidobacterium* would also explain the increase in predicted gene counts for oligosaccharide reducing-end xylanase in the cecal contents, cecal mucosa, and colonic mucosa (Grootaert et al., 2007; Moura et al., 2007). Moreover, *Bifidobacterium* have the capacity to metabolize pentoses through the bifid shunt pathway which ultimately results in the production of acetate (Rivière et al., 2016). This would explain the increase in cecal acetate production observed by Petry et al. (2020b), and the rise in *Faecalibacterium* in the cecal mucosa. *Faecalibacterium*, whose only known species is *Faecalibacterium prausnitzii*, is an acetate-consuming and butyrate-producing bacterium that does not utilize carbohydrates efficiently and requires acetate for optimal proliferation (Falony et al., 2006; Miquel et al., 2014). The increase in *Faecalibacterium* in colonic contents by HF+XY may also explain the increase in molar proportion of butyrate reported by Petry et al. (2020b).

Butyrate is the preferential energy source for colonocytes and is a potent modulator of gastrointestinal health (Bedford and Gong, 2018). Moreover, butyrate aids in the control of enteric pathogens, reduces inflammation, and mitigates reactive oxygen species (Bach Knudsen et al., 2018). The butyrate produced from the metabolic cross-feeding of AXOS by *Lactobacillus*, *Bifidobacterium*, and *Faecalibacterium* may potentially explain the reductions in finishing pig mortality often observed when xylanase is used in commercial pork production (Zier-Rush et al., 2016). Moreover, in recent years, there have been several studies that observed xylanase modulates gastrointestinal health, immune function, oxidative stress, and intestinal morphology of pigs (Tiwari et al., 2018; Chen et al., 2020; Petry et al., 2020b). Potentially, these health benefits are a consequence of the aforementioned microbial modulation by xylanase.

## Conclusion

Diets with higher DF increase microbial diversity in the large intestine and favor microbial communities that degrade complex carbohydrates and mucins. However, HF also reduced microbial communities found in abundance in the microbiome of more feed efficient pigs. Supplementing xylanase in HF further increased microbial diversity in cecal contents and colonic mucosa and favored microbial communities that produce microbial carbohydrases and ferment AXOS. Of particular interest, HF+XY increased the abundance of *Lactobacillus*, *Bifidobacterium*, and *Faecalibacterium* across the large intestine. These genera are known to work cooperatively to produce butyrate and may explain the unexpected health benefits commonly observed with xylanase supplementation in pigs. These data support the recently proposed stimbiotic MOA of xylanase. Compared to xylanase, AXOS did not elicit a similar response in hindgut microbiota. It is likely this oligosaccharide differs in composition from that produced by xylanase or may have been fermented in the small intestine. These data warrant further investigation into the stimbiotic potential of xylanase.

## Data Availability Statement

The datasets presented in this study can be found in online repositories. The name of the repository and accession number can be found below: National Center for Biotechnology Information (NCBI) BioProject, https://www.ncbi.nlm.nih.gov/, PRJNA667518.

## Ethics Statement

The animal study was reviewed and approved by the Institutional Animal Care and Use Committee at Iowa State University (IACUC #9-17-8613-S), and followed the guidelines for the ethical and humane use of animals for research according to the Guide for the Care and Use of Agricultural Animals in Research and Teaching (FASS, 2010).

## Author Contributions

AP, NH, MB, and JP designed the experiment. AP performed the experiment, collected data, and conducted DNA extractions. AP and LK performed the bioinformatics and statistical analysis of the 16S rDNA sequencing data. SS-E contributed to the concept of microbial sequencing data analysis and results interpretation. JP was the principal investigator, who supervised all aspects of the study. All authors contributed to the article and approved the submitted version.

### Conflict of Interest

The authors declare that this study received funding from AB Vista. The funder had the following involvement with the study: AB Vista was involved in the conceptualization of the project and study design, and they provided the xylanase and AXOS used in this trial. MB was employed by AB Vista and contributed to the study design, and had a minor role in the decision to publish and the preparation of the manuscript.

The remaining authors declare that the research was conducted in the absence of any commercial or financial relationships that could be construed as a potential conflict of interest.
