# Supplementary material for: Xylanase Supplementation Modulates the Microbiota of the Large Intestine of Pigs Fed Corn-Based Fiber by Means of a Stimbiotic Mechanism of Action
Source: Front Microbiol. 2021 Mar 24;12:619970. doi: 10.3389/fmicb.2021.619970 (PMC8024495; doi:10.3389/fmicb.2021.619970)
Supplement: Supplementary file 1 [file Data_Sheet_1.pdf]

Supplmentary Material

Supplmentary Figure 1. Summary of phyla, family, and genera microbial composition of cecal contents and mucosa among all pigs (N=60). (**A)** Phyla composistion of cecal contents; (**B**) Family composistion of cecal contents; (**C**); Genera composistion of cecal contents; (**D)** Phyla composistion of cecal mucosa; (**E**) Family composistion of cecal mucosa; (**F**); Genera composistion of cecal mucosa.

Supplmentary Figure 2. Summary of phyla, family, and genera microbial composition of colonic contents and mucosa among all pigs (N=60). (**A)** Phyla composistion of colonic contents; (**B**) Familiae composistion of colonic contents; (**C**); Genera composistion of colonic contents; (**D)** Phyla composistion of colonic mucosa; (**E**) Family composistion of colonic mucosa; (**F**); Genera composistion of colonic mucosa.

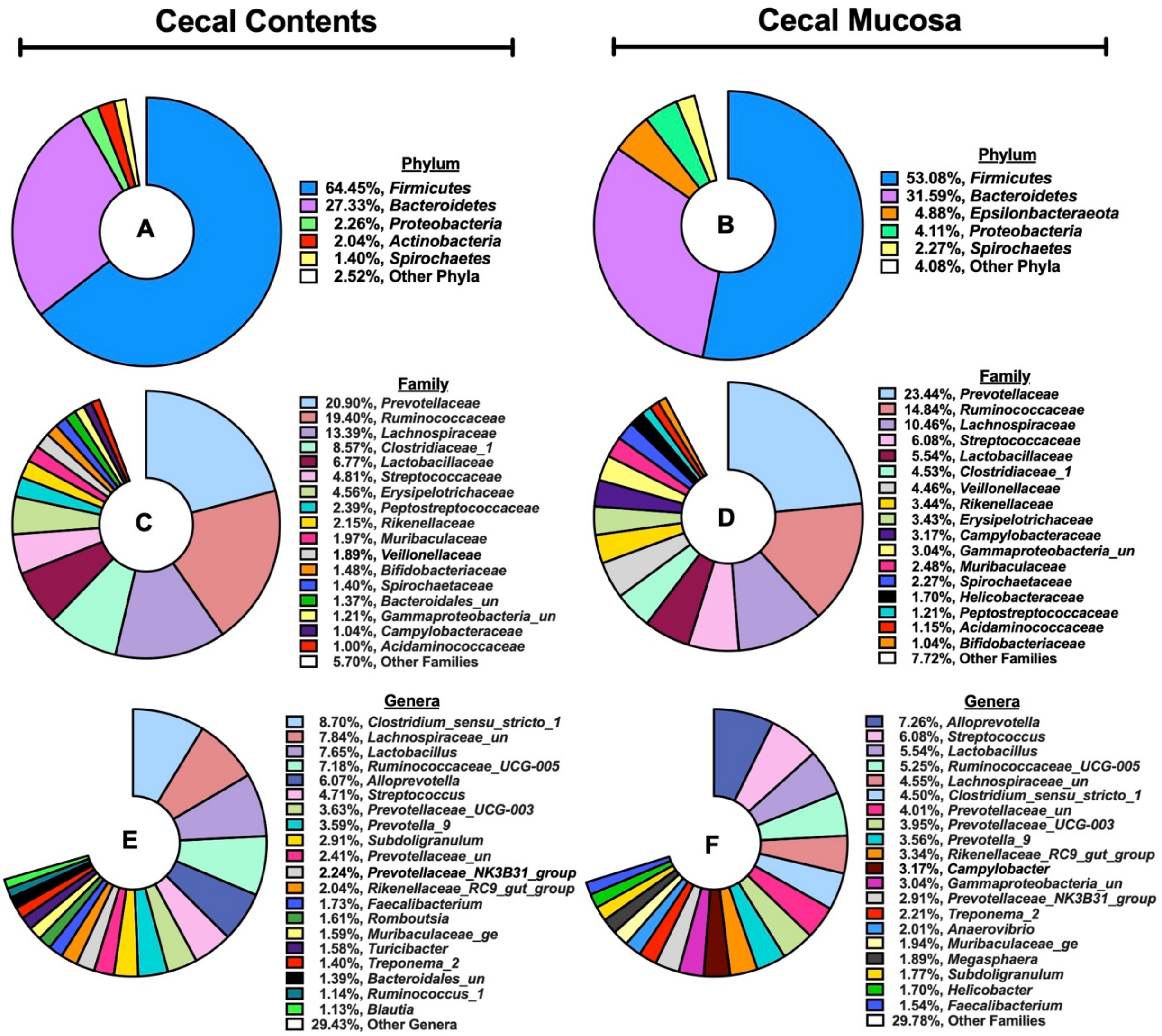
Cecal Contents
Cecal Mucosa
A
Phylum
64.45%, Firmicutes
27.33%, Bacteroidetes
2.26%, Proteobacteria
2.04%, Actinobacteria
1.40%, Spirochaetes
2.52%, Other Phyla
B
Phylum
53.08%, Firmicutes
31.59%, Bacteroidetes
4.88%, Epsilonbacteraeota
4.11%, Proteobacteria
2.27%, Spirochaetes
4.08%, Other Phyla
C
Family
20.90%, Prevotellaceae
19.40%, Ruminococcaceae
13.39%, Lachnospiraceae
8.57%, Clostridiaceae_1
6.77%, Lactobacillaceae
4.81%, Streptococcaceae
4.56%, Erysipelotrichaceae
2.39%, Peptostreptococcaceae
2.15%, Rikenellaceae
1.97%, Muribaculaceae
1.89%, Veillonellaceae
1.48%, Bifidobacteriaceae
1.40%, Spirochaetaceae
1.37%, Bacteroidales_un
1.21%, Gammaproteobacteria_un
1.04%, Campylobacteraceae
1.00%, Acidaminococcaceae
5.70%, Other Families
D
Family
23.44%, Prevotellaceae
14.84%, Ruminococcaceae
10.46%, Lachnospiraceae
6.08%, Streptococcaceae
5.54%, Lactobacillaceae
4.53%, Clostridiaceae_1
4.46%, Veillonellaceae
3.44%, Rikenellaceae
3.43%, Erysipelotrichaceae
3.17%, Campylobacteraceae
3.04%, Gammaproteobacteria_un
2.48%, Muribaculaceae
2.27%, Spirochaetaceae
1.70%, Helicobacteraceae
1.21%, Peptostreptococcaceae
1.15%, Acidaminococcaceae
1.04%, Bifidobacteriaceae
7.72%, Other Families
E
Genera
8.70%, Clostridium_sensu_stricto_1
7.84%, Lachnospiraceae_un
7.65%, Lactobacillus
7.18%, Ruminococcaceae_UCG-005
6.07%, Alloprevotella
4.71%, Streptococcus
3.63%, Prevotellaceae_UCG-003
3.59%, Prevotella_9
2.91%, Subdoligranulum
2.41%, Prevotellaceae_un
2.24%, Prevotellaceae_NK3B31_group
2.04%, Rikenellaceae_RC9_gut_group
1.73%, Faecalibacterium
1.61%, Romboutsia
1.59%, Muribaculaceae_ge
1.58%, Turicibacter
1.40%, Treponema_2
1.39%, Bacteroidales_un
1.14%, Ruminococcus_1
1.13%, Blautia
29.43%, Other Genera
F
Genera
7.26%, Alloprevotella
6.08%, Streptococcus
5.54%, Lactobacillus
5.25%, Ruminococcaceae_UCG-005
4.55%, Lachnospiraceae_un
4.50%, Clostridium_sensu_stricto_1
4.01%, Prevotellaceae_un
3.95%, Prevotellaceae_UCG-003
3.56%, Prevotella_9
3.34%, Rikenellaceae_RC9_gut_group
3.17%, Campylobacter
3.04%, Gammaproteobacteria_un
2.91%, Prevotellaceae_NK3B31_group
2.21%, Treponema_2
2.01%, Anaerovibrio
1.94%, Muribaculaceae_ge
1.89%, Megasphaera
1.77%, Subdoligranulum
1.70%, Helicobacter
1.54%, Faecalibacterium
29.78%, Other Families

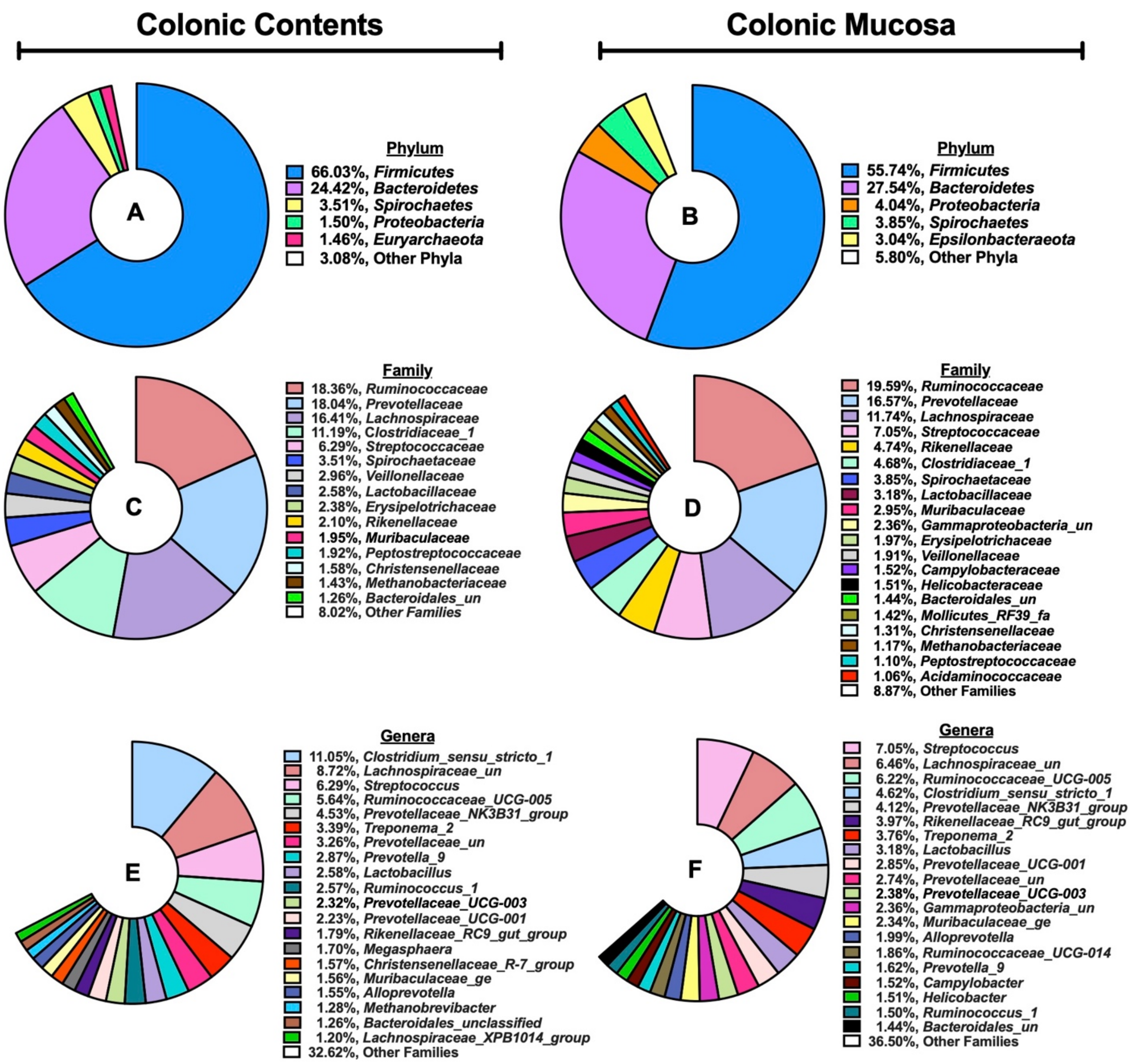
Colonic Contents
Colonic Mucosa
A
Phylum
66.03%, Firmicutes
24.42%, Bacteroidetes
3.51%, Spirochaetes
1.50%, Proteobacteria
1.46%, Euryarchaeota
3.08%, Other Phyla
B
Phylum
55.74%, Firmicutes
27.54%, Bacteroidetes
4.04%, Proteobacteria
3.85%, Spirochaetes
3.04%, Epsilonbacteraeota
5.80%, Other Phyla
C
Family
18.36%, Ruminococcaceae
18.04%, Prevotellaceae
16.41%, Lachnospiraceae
11.19%, Clostridiaceae_1
6.29%, Streptococcaceae
3.51%, Spirochaetaceae
2.96%, Veillonellaceae
2.58%, Lactobacillaceae
2.38%, Erysipelotrichaceae
2.10%, Rikenellaceae
1.95%, Muribaculaceae
1.92%, Peptostreptococcaceae
1.58%, Christensenellaceae
1.43%, Methanobacteriaceae
1.26%, Bacteroidales_un
8.02%, Other Families
D
Family
19.59%, Ruminococcaceae
16.57%, Prevotellaceae
11.74%, Lachnospiraceae
7.05%, Streptococcaceae
4.74%, Rikenellaceae
4.68%, Clostridiaceae_1
3.85%, Spirochaetaceae
3.18%, Lactobacillaceae
2.95%, Muribaculaceae
2.36%, Gammaproteobacteria_un
1.97%, Erysipelotrichaceae
1.91%, Veillonellaceae
1.52%, Campylobacteraceae
1.51%, Helicobacteraceae
1.44%, Bacteroidales_un
1.42%, Mollicutes_RF39_fa
1.31%, Christensenellaceae
1.17%, Methanobacteriaceae
1.10%, Peptostreptococcaceae
1.06%, Acidaminococcaceae
8.87%, Other Families
E
Genera
11.05%, Clostridium_sensu_stricto_1
8.72%, Lachnospiraceae_un
6.29%, Streptococcus
5.64%, Ruminococcaceae_UCG-005
4.53%, Prevotellaceae_NK3B31_group
3.39%, Treponema_2
3.26%, Prevotellaceae_un
2.87%, Prevotella_9
2.58%, Lactobacillus
2.57%, Ruminococcus_1
2.32%, Prevotellaceae_UCG-003
2.23%, Prevotellaceae_UCG-001
1.79%, Rikenellaceae_RC9_gut_group
1.70%, Megasphaera
1.57%, Christensenellaceae_R-7_group
1.56%, Muribaculaceae_ge
1.55%, Alloprevotella
1.28%, Methanobrevibacter
1.26%, Bacteroidales_unclassified
1.20%, Lachnospiraceae_XPB1014_group
32.62%, Other Families
F
Genera
7.05%, Streptococcus
6.46%, Lachnospiraceae_un
6.22%, Ruminococcaceae_UCG-005
4.62%, Clostridium_sensu_stricto_1
4.12%, Prevotellaceae_NK3B31_group
3.97%, Rikenellaceae_RC9_gut_group
3.76%, Treponema_2
3.18%, Lactobacillus
2.85%, Prevotellaceae_UCG-001
2.74%, Prevotellaceae_un
2.38%, Prevotellaceae_UCG-003
2.36%, Gammaproteobacteria_un
2.34%, Muribaculaceae_ge
1.99%, Alloprevotella
1.86%, Ruminococcaceae_UCG-014
1.62%, Prevotella_9
1.52%, Campylobacter
1.51%, Helicobacter
1.50%, Ruminococcus_1
1.44%, Bacteroidales_un
36.50%, Other Families
